# Association of household wealth and education with hypertension among adults in Lesotho: Evidence from a propensity score–based study

**DOI:** 10.1371/journal.pgph.0006599

**Published:** 2026-06-10

**Authors:** Rajat Das Gupta, Amrit Baral, Shams Shabab Haider, Promit Ananyo Chakraborty

**Affiliations:** 1 Division of Epidemiology, Department of Medicine, Vanderbilt University Medical Center, Nashville, Tennessee, United States of America; 2 Department of Epidemiology and Biostatistics, Arnold School of Public Health, University of South Carolina, Columbia, South Carolina, United States of America; 3 Department of Mental Health, Bloomberg School of Public Health, Johns Hopkins University, Baltimore, Maryland, United States of America; 4 Health Data Management Project, Friendship NGO, Dhaka, Bangladesh; 5 Department of Social Relations, East West University, Dhaka, Bangladesh; 6 School of Population and Public Health, University of British Columbia, Vancouver, British Columbia, Canada; Universitas Muhammadiyah Aceh, INDONESIA

## Abstract

In Lesotho, rapid urbanization and lifestyle transitions have contributed to a rising burden of hypertension, yet the role of socioeconomic factors remains inadequately understood. This study examined the independent and joint associations of household wealth and educational attainment with hypertension among adults in Lesotho. We analyzed data from 5,457 adults aged 18–49 years from the 2023–2024 Lesotho Demographic and Health Survey. Hypertension was defined as systolic blood pressure ≥140 mmHg, diastolic blood pressure ≥90 mmHg, self-reported prior diagnosis by a health professional, or current use of antihypertensive medication. The main exposures were household wealth status (poor vs non poor) and educational attainment (below secondary vs secondary or higher). Propensity scores were estimated using a super learner algorithm and applied through one-to-one nearest neighbor matching (PS matched) and inverse probability weighting (IPW) to adjust for covariates. Weighted logistic regression models generated adjusted odds ratios (AORs) and 95% confidence intervals (CIs). Overall, 21.2% (weighted) of participants were hypertensive. Adults from non poor households had significantly higher odds of hypertension than those from poor households (PS matched AOR 1.56, 95% CI 1.13–2.14; IPW AOR 1.76, 95% CI 1.21–2.57). Educational attainment alone was not significantly associated with hypertension. The wealth related gradient persisted across education strata, with the highest odds observed among non poor adults with secondary or higher education (AOR 2.06, 95% CI 1.34–3.16). Household wealth, but not education, was independently associated with hypertension among adults in Lesotho.

## Introduction

Hypertension is a leading cardiovascular risk factor, contributing to nearly five million deaths each year [1]. An estimated 1.3 billion adults live with hypertension, making it the top global driver of death and disability. Prevalence varies across regions, affecting about 28% of adults in South Asia and up to 46% in Africa, where uncontrolled blood pressure accounts for a substantial share of premature mortality [2]. Overall, more than three-quarters of people with hypertension live in low- and middle-income countries, with Africa being disproportionately affected [3].

In Lesotho, hypertension has emerged as a significant public health concern, with surveys reporting prevalence estimates ranging from 21% to 31% among adults [4,5]. High salt intake, tobacco use, obesity, alcohol consumption, and physical inactivity contribute to a high hypertension burden in Lesotho [6]. Importantly, treatment coverage and control remain low, leaving many individuals at risk of complications such as stroke and cardiovascular disease [7]. Understanding how household wealth and educational attainment influence disease distribution can help inform the design of equitable prevention and control strategies that are both efficient and contextually appropriate for Lesotho.

Socioeconomic status (SES) is a powerful determinant of health, but its relationship with hypertension varies across settings. In high-income countries, lower SES, reflected in reduced income, wealth, or education, is consistently associated with higher hypertension prevalence due to barriers in healthcare access, increased psychosocial stress, and cumulative disadvantage [8]. In low- and middle-income countries (LMICs), however, the pattern is less straightforward [9]. Studies suggest that in the context of rapid urbanization and dietary transition, wealthier individuals may have higher hypertension risk due to sedentary lifestyles, consumption of energy-dense foods, and rising obesity [10,11].

Evidence from South Asia illustrates this complexity. In Bangladesh, Das Gupta et al. (2021) found that household wealth was significantly associated with diabetes but not with hypertension, and that education was not associated with either outcome [12]. In India, Filmer and Pritchett demonstrated that wealth indices strongly predict education outcomes [13]; however, their health implications, particularly hypertension, remain context-dependent. In African settings, results are also mixed. A study in Ghana observed that women from higher socioeconomic backgrounds were more likely to have hypertension, attributed to urban residence and obesity [14]. In contrast, others reported that disadvantaged groups bore greater risks [15,16]. Collectively, these findings underscore the need to contextualize the SES-hypertension relationship within local social, cultural, and economic environments.

Despite the growing recognition of hypertension as a leading cause of morbidity in Lesotho, few studies have examined how wealth and education influence its burden. Previous national surveys have described the prevalence [17], but have not accounted for the confounding and interaction effects of SES. Moreover, to our knowledge, no study has applied advanced causal-analytic techniques, such as propensity score-based methods, to disentangle the independent and combined effects of wealth and education on hypertension in Lesotho.

The limited evidence on how socioeconomic determinants shape hypertension risk in Lesotho represents a critical gap. Without this knowledge, prevention and control strategies risk being poorly targeted, overlooking the most vulnerable groups, or misdirecting scarce health resources. This concern is particularly relevant in Lesotho, where stark inequalities in wealth and education exist and the health system is already strained by both communicable and noncommunicable diseases.

The objective of this secondary analysis is to investigate the association between household wealth, educational attainment, and hypertension among adults in Lesotho using data from the 2023–2024 Lesotho Demographic and Health Survey (LDHS 2023–2024). Specifically, we aim to (1) estimate the independent associations of household wealth and education with hypertension, and (2) examine whether these factors interact to influence hypertension risk jointly.

### Conceptual framework

Guided by established frameworks on social determinants of health, household wealth and educational attainment were conceptualized as distinct dimensions of socioeconomic status that influence hypertension through multiple pathways [18]. Household wealth reflects material resources and living conditions, shaping access to food environments, healthcare services, and patterns of urban residence, while educational attainment is associated with health literacy, behavioral capacity, and psychosocial resources such as perceived control and the ability to manage stress. These influences may operate through behavioral pathways (e.g., diet, physical activity, tobacco and alcohol use) and psychosocial processes, including chronic stress and social comparison, which can affect blood pressure through neuroendocrine mechanisms [18].

**Fig 1:**
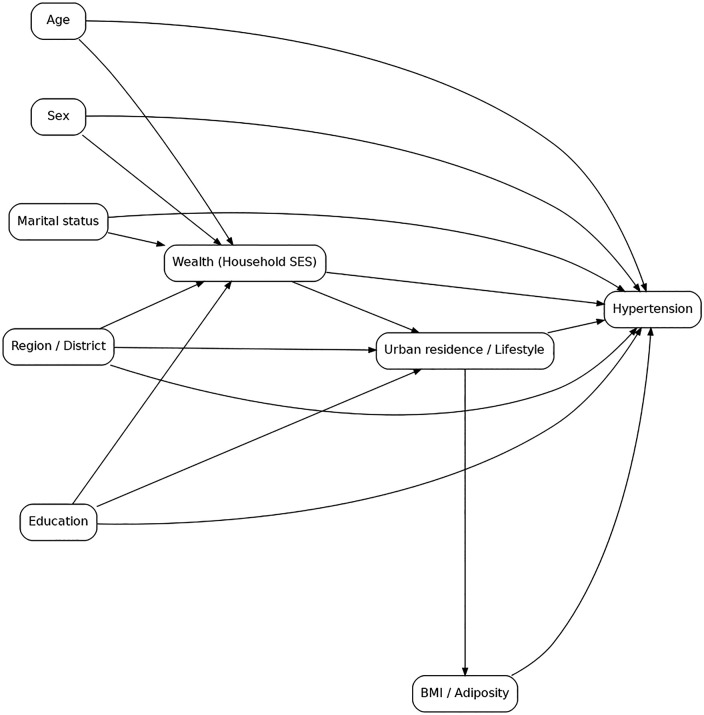
Conceptual framework illustrating the relationships between socioeconomic factors and hypertension in Lesotho.

In the present study, this framework was operationalized using available LDHS covariates, including age, sex, marital status, body mass index (BMI), ecological zone, region of residence, and place of residence [12,19] (Fig 1). These variables were selected as potential confounders representing demographic, geographic, and anthropometric factors associated with both socioeconomic status and hypertension risk.

Although important behavioral determinants such as dietary intake and physical activity were not available in the dataset, these factors are likely to lie on the causal pathway linking socioeconomic status to hypertension and were therefore not explicitly modeled but are considered in the interpretation of the findings. Differences in access to healthcare and diagnosis may also contribute to observed disparities.

Within this framework, household wealth and educational attainment may operate both independently and jointly to influence hypertension risk. Their interaction may reflect how material resources and health-related knowledge combine within the sociocultural context of Lesotho to shape hypertension risk.

## Methods

### Ethics considerations

Ethical clearance for the LDHS 2023–2024 was granted by the ICF Institutional Review Board (Approval No. 2023–150) and the National Health Research Ethics Committee of the Lesotho Ministry of Health (Approval No. ID193–2023). All participants provided written informed consent before taking part in the survey and biomarker assessments. Informed consent was obtained from all participants prior to the original data collection. For the present secondary analysis, access was granted to anonymized deidentified datasets through the DHS Program following approval of our research proposal on August 13, 2025. Because the analyses were conducted exclusively using de-identified data, additional ethical review or approval was not required.

### Study design and data source

This study used data from the LDHS 2023–2024, the fourth round of the Demographic and Health Survey (DHS) conducted in the country. The survey was implemented by the Ministry of Health in collaboration with the Lesotho Bureau of Statistics, with technical assistance from ICF through the DHS Program and financial support from the United States Agency for International Development (USAID). Data collection took place from 27 November 2023 to 29 February 2024. The LDHS 2023–2024 was designed to provide nationally representative estimates of demographic and health indicators, including fertility, reproductive health, maternal and child health, nutrition, infectious and chronic diseases, noncommunicable diseases (NCDs), and gender-based violence. Detailed descriptions of the study methodology and survey design have been published elsewhere [19].

The LDHS 2023–2024 followed a two-stage stratified sampling procedure using the 2016 Lesotho Population and Housing Census as the sampling frame. In the first stage, 400 enumeration areas were selected with probability proportional to size from 29 strata defined by district and urban, peri-urban, or rural residence (with Butha-Buthe district not including peri-urban areas). In the second stage, 25 households were systematically sampled from each enumeration area. All women aged 15–49 years were eligible to participate in the Women’s Questionnaire, while men aged 15–59 years were eligible in half of the selected households. In this subsample of households, biomarker measurements, including blood pressure, were collected for all eligible adults. Fieldwork was carried out by 15 trained teams, and data were collected electronically using tablet-based questionnaires [19].

### Study population

For this analysis, we included adults aged 18–49 years who had valid blood pressure measurements. Although individuals aged 15–17 years were eligible in the original DHS sample, we restricted the analysis to adults to ensure epidemiological and clinical comparability, as hypertension definitions, risk profiles, and physiological characteristics differ between adolescents and adults. Pregnant women and participants with missing values for key covariates were excluded (<1% of the eligible sample). After exclusions, the final analytic sample consisted of 5,457 adults.

### Outcome variable

The outcome of interest was hypertension status. Trained biomarker technicians measured blood pressure using calibrated automated devices. Three measurements were taken at 10-minute intervals, and the average of the second and third readings was used [19]. Hypertension was defined according to the 2003 World Health Organization/International Society of Hypertension (WHO/ISH) statement and the DHS program as systolic blood pressure of at least 140 mmHg and/or diastolic blood pressure of at least 90 mmHg. Individuals were also classified as hypertensive if they reported having ever been diagnosed with the condition by a health professional or if they were currently taking prescribed antihypertensive medication [19,20]. The variable was analyzed as a binary outcome (hypertension versus no hypertension).

### Exposure variables

The primary exposures were household wealth status and educational attainment. Household wealth status, categorized as poor (poorest and poorer quintiles) or non-poor (middle, richer, and richest quintiles). The DHS constructs the wealth index using principal component analysis based on household assets and amenities [13,19,21]. Educational attainment, dichotomized as no secondary/higher education (none or primary) versus secondary/higher education.

### Covariates

We considered a set of potential confounders based on prior literature [12] and the DHS conceptual framework [19]. These included age group (18–29, 30–39, 40–49 years), sex (male, female), marital status (never married, married, widowed/divorced/separated), BMI (underweight, normal, overweight, obese), place of residence (urban, rural), ecological zone (lowlands, foothills, mountains, Senqu River Valley), and region of residence (10 administrative districts) [19].

### Statistical analysis

All statistical analyses were conducted using Stata 19.0 (StataCorp LLC, College Station, TX). All analyses accounted for the complex survey design, including sampling weights, clusters, and strata, using the *“svy”* command of Stata. The sample characteristics were first described using weighted percentages and assessed bivariate associations between covariates and hypertension with Rao-Scott Chi-square tests adjusted for design effects [22].

For the main analysis, we applied propensity score (PS) methods to estimate the association of household wealth and education with hypertension [23]. Separate PS models were constructed for each exposure. When household wealth status was treated as exposure, educational attainment was included as a covariate in the PS model. Conversely, when educational attainment was treated as exposure, household wealth status was included as a covariate. In both cases, PS were estimated using a logistic model incorporating age, sex, marital status, body mass index, ecological zone, region of residence, and place of residence, along with survey design variables (strata and cluster), to control for confounding.

Two PS approaches were applied: (1) 1:1 nearest neighbor matching without replacement within a caliper of 0.2, to match non-poor with poor individuals and those with versus without secondary/higher education [24]. PS were generated using a super learner algorithm with fivefold cross-validation, which was applied to enhance covariate balance and minimize the risk of model misspecification [25]. A standardized mean difference (SMD) of 0.1 or less was taken to indicate adequate covariate balance [26]. Design-based binary logistic regression models were then fitted to the matched samples using survey-adjusted (“svy”) procedures. The regression models were adjusted for all covariates included in the propensity score estimation to address any remaining imbalance between comparison groups [27].

Second, inverse probability weighting (IPW) based on the estimated propensity scores was applied. Stabilized inverse probability weights were derived from the PS models and multiplied by the original survey sampling weights to obtain population-representative estimates [23]. Weighted outcome models were estimated using survey-adjusted logistic regression.

Separate models were fitted for wealth status and educational attainment. For the wealth analysis, individuals from non-poor households were compared with those from poor households; for the education analysis, adults with secondary or higher education were compared with those with lower education.

To evaluate the robustness of the propensity score matching findings, additional sensitivity analyses were performed using design-based logistic regression models [28]. In the first step, multivariable logistic regression was estimated while adjusting for the same covariates included in the PS models and accounting for the survey design. In the second step, an interaction term between household wealth status and educational attainment was introduced into the model to examine potential effect modification. Adjusted odds ratios (AORs) with 95% confidence intervals (CIs) were reported. Statistical significance was defined as *p* < 0.05.

## Findings

### Characteristics of the study population

**Fig 2:**
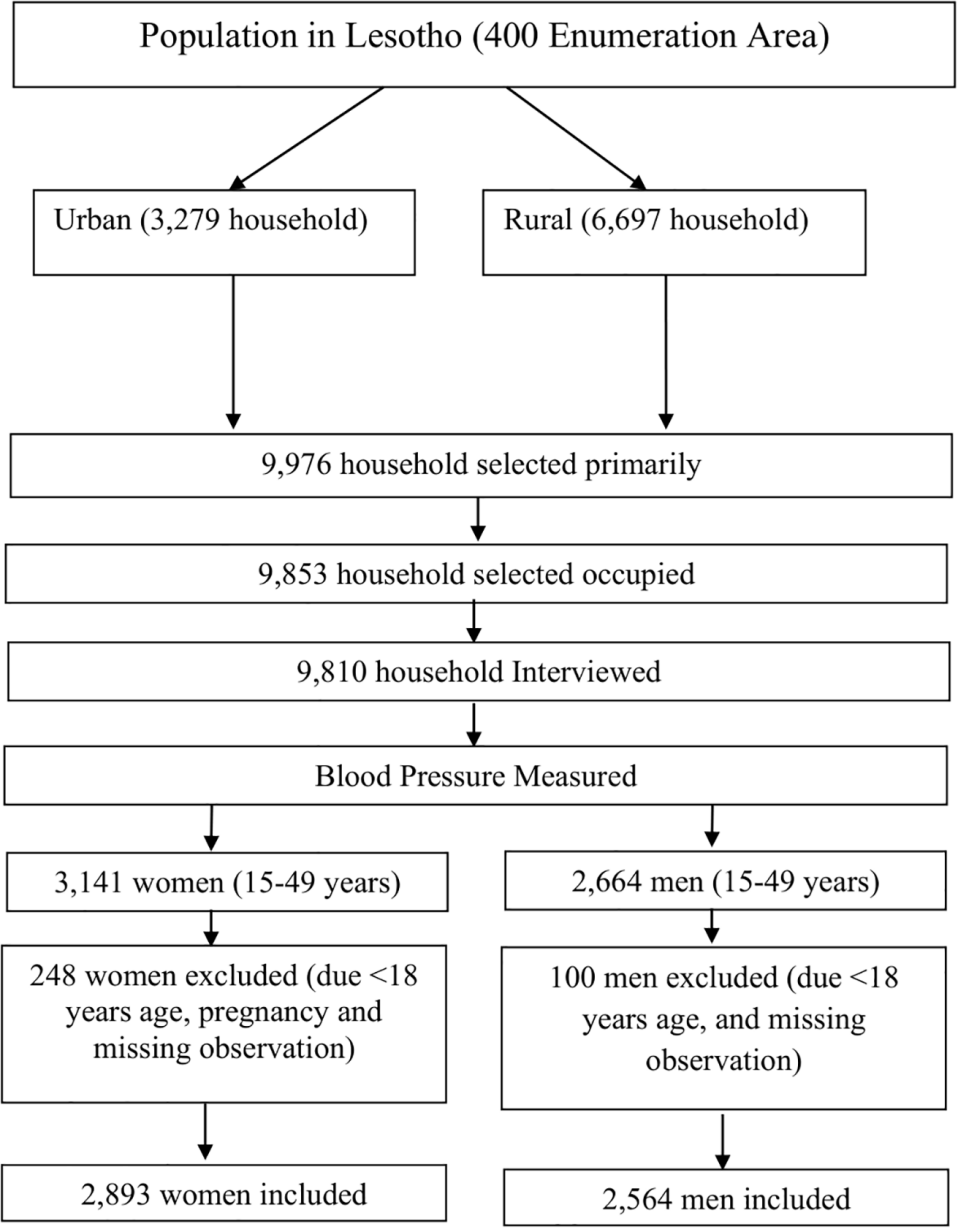
Flowchart illustrating the process of analytic sample selection.

Table 1 presents the characteristics of 5,457 adults aged 18–49 years in Lesotho, stratified by hypertension status. **Fig 2** shows the flowchart of analytic sample selection. Overall, 21.2% of participants were hypertensive. Hypertension was significantly more common among adults from non-poor households (25.0%) compared with those from poor households (13.1%, p < 0.001). In contrast, the prevalence of hypertension did not differ significantly by education level (p > 0.05).

**Table 1:** Characteristics of the study sample by hypertension status among 18-49-year-old adults in the Lesotho Demographic and Health Survey 2023-24 (N = 5,457).

Characteristics	N (Unweighted)	% (Weighted)	Hypertension (%)	*p*-value
**Hypertension**	964	21.21	–	
**Wealth Index**				<0.001
Poor	2406	31.76	13.1	
Non-poor	3051	68.24	24.99	
**Education**				>0.05
No education/primary education	2183	33.72	19.8	
Secondary/higher education	3274	66.28	21.93	
**Age in years**				<0.001
18-29	2490	45.45	11.91	
30-39	1664	29.82	25.11	
40-49	1303	24.73	33.62	
**Sex**				>0.05
Male	2564	46.83	20.24	
Female	2893	53.17	22.07	
**Marital Status**				<0.001
Never married	2007	37.85	14.83	
Married	2741	49.38	24.61	
Widowed/Divorced/Separated	709	12.77	26.98	
**Ecological Zone**				<0.001
Lowlands	2872	72.32	24.36	
Foothills	419	7.22	12	
Mountains	1469	14.61	12.63	
Senqu River Valley	697	5.85	15.14	
**Region of Residence**				<0.001
Butha-Buthe	574	5.97	14.48	
Leribe	691	18.19	18.16	
Berea	642	15	24.75	
Maseru	750	33.99	27.44	
Mafeteng	498	6.29	21.14	
Mohale’s Hoek	419	4.44	12.21	
Quthing	449	3.46	20.73	
Qacha’s Nek	400	2.76	10.23	
Mokhotlong	488	3.99	16.78	
Thaba-Tseka	546	5.91	7.93	
**Place of Residence**				<0.001
Urban	1978	44.26	27.91	
Rural	3479	55.74	15.9	
**BMI Categories**				<0.001
Underweight	704	15.2	48.61	
Normal BMI	2772	48.16	9.08	
Overweight	1029	18.77	20.27	
Obesity	952	17.87	31.61	

BMI: Body Mass Index; the frequencies are unweighted, while the percentages represent weighted estimates.

Age showed a strong gradient with hypertension prevalence, rising from 11.9% in those aged 18–29 years to 33.6% in the 40–49 age group (p < 0.001). Hypertension prevalence was slightly higher among women (22.1%) than men (20.2%), though this difference was not statistically significant (p > 0.05). Marital status was also associated with hypertension, with the highest prevalence observed among widowed, divorced, or separated individuals (27.0%, p < 0.001).

In ecological zones, adults residing in the Lowlands had the highest prevalence of hypertension (24.4%), followed by those in the Foothills (12.0%) and Mountains (12.6%, p < 0.001). At the regional level, prevalence ranged from 7.9% in Thaba-Tseka to 27.4% in Maseru (p < 0.001). Urban residents had a higher prevalence of hypertension compared to their rural counterparts (27.9% vs 15.9%, p < 0.001).

BMI was strongly associated with hypertension. Notably, almost half (48.6%) of underweight individuals were hypertensive, compared with 9.1% of those with normal BMI, 20.3% of those overweight, and 31.6% of those obese (p < 0.001).

### Propensity score analyses

**Fig 3:**
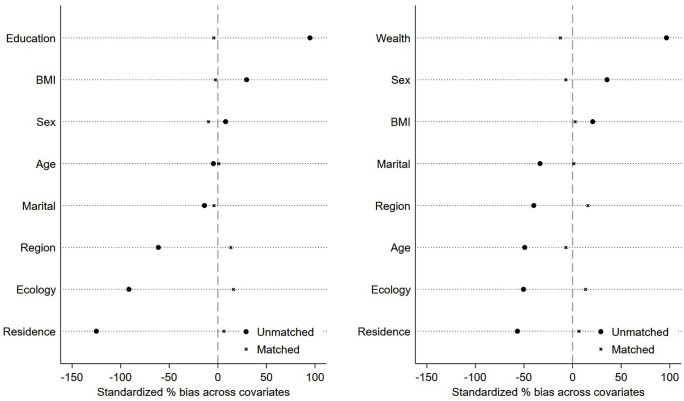
Standardized mean differences (SMDs) for wealth status (poor vs non-poor, left panel) and educational attainment (no secondary/higher vs secondary/higher education, right panel) are shown for both unmatched and propensity score–matched samples. Following matching, covariate balance was achieved, with nearly all SMDs reduced to within the 0.10 threshold. BMI refers to body mass index.

**Fig 3** displays the SMDs for wealth status (left panel) and education (right panel) in the unmatched and propensity score–matched samples. Among 3,051 adults from non-poor households, 2,406 were successfully matched to adults from poor households (unweighted data). Although several covariates showed considerable imbalance before matching, the procedure substantially improved comparability, with most SMDs reduced to below or close to the 0.1 threshold [12]. For education, 2,183 adults without secondary/higher education were matched with an equal number of adults with secondary/higher education, yielding 2,183 matched pairs. Similar to wealth, matching notably reduced covariate imbalance, and overall balance was deemed acceptable.

**Table 2** presents the PS based analyses. After 1:1 matching, adults from non-poor households had significantly higher odds of hypertension than those from poor households (AOR: 1.56, 95% CI: 1.13–2.14). The association was even stronger in the inverse probability weighting analysis (AOR: 1.76, 95% CI: 1.21–2.57). On the other hand, education status was not significantly associated with hypertension in either approach.

**Table 2:** Association of wealth status and education level with hypertension among 18-49 years old adults in Lesotho using the propensity score approaches.

Propensity score method	Exposure of Interest	Hypertension	
		AOR	95% CI
Matching	**Household wealth status**		
	Poor	Ref	
	Non-poor	1.56	1.13–2.14
	**Secondary/higher education**		
	No	Ref	
	Yes	1.10	0.82–1.47
Weighting	**Household wealth status**		
	Poor	Ref	
	Non-poor	1.76	1.21–2.57
	**Secondary/higher education**		
	No	Ref	
	Yes	1.07	0.82–1.39

AOR: Adjusted Odds Ratio; CI: Confidence Interval; Ref: Reference.The outcome model was adjusted for age, sex, marital status, body mass index, ecological region, region of residence, place of residence, education (not considered in the secondary/higher education-hypertension relationship), and household wealth status (not considered in the non-poor-hypertension relationship).

**Table 3** shows the interaction between wealth and education in relation to hypertension. Among individuals with no secondary/higher education, those from non-poor households were more likely to have hypertension than those from poor households (AOR: 1.46, 95% CI: 1.02–2.08). The association was even stronger among those with secondary/higher education (AOR: 1.64, 95% CI: 1.19–2.24). Analyses with PS weighting produced similar results, with the odds of hypertension being more than twofold higher among non-poor adults with secondary/higher education compared to their poor counterparts (AOR: 2.06, 95% CI: 1.34–3.16). Education itself was not independently associated with hypertension within either wealth stratum.

**Table 3:** Interaction effects of wealth status and education level with hypertension among 18-49 years old adults in Lesotho using the propensity score approaches.

Propensity score method	Exposure of Interest	Hypertension	
		AOR	95% CI
Matching	**Household wealth status × Education**		
	Non-poor (*vs*. Poor) within No secondary/higher education	1.46	1.02–2.08
	Non-poor (*vs*. Poor) within Secondary/higher education	1.64	1.19–2.24
	Secondary/higher education (Yes *vs*. No) within Poor	0.97	0.68–1.40
	Secondary/higher education (Yes *vs*. No) within Non-poor	1.12	0.48–2.60
Weighting	**Household wealth status × Education**		
	Non-poor (*vs*. Poor) within No secondary/higher education	1.74	1.08–2.80
	Non-poor (*vs*. Poor) within Secondary/higher education	2.06	1.34–3.16
	Secondary/higher education (Yes *vs*. No) within Poor	0.82	0.40–1.68
	Secondary/higher education (Yes *vs*. No) within Non-poor	1.18	0.26–5.31

AOR: Adjusted Odds Ratio; CI: Confidence Interval; Ref: Reference.

The outcome model was adjusted for age, sex, marital status, body mass index, ecological region, region of residence, and place of residence.

### Sensitivity analyses

**Table 4** presents the design-based logistic regression results, which were consistent with the PS analyses. Non-poor adults remained at significantly higher odds of having hypertension compared with poor adults (AOR: 1.60, 95% CI: 1.22–2.11). Education level was not significantly associated with hypertension. Interaction terms showed a consistent positive association between wealth and hypertension across both education categories, with stronger effects observed among those with secondary/higher education.

**Table 4:** Sensitivity analyses for the association of wealth status and education level with hypertension among 18-49 years old adults in Lesotho using the design-based logistic regression.

Variables	Level / Contrast	AOR	95% CI
Household wealth status	Poor	Ref.	
Household wealth status	Non-poor	1.60	1.22–2.11
Secondary/higher education	No	Ref.	
Secondary/higher education	Yes	1.19	0.93–1.52
Household wealth status × Education	Non-poor (vs. Poor) within No secondary/higher education	1.46	1.02–2.08
Household wealth status × Education	Non-poor (vs. Poor) within Secondary/higher education	1.68	1.14–2.48
Household wealth status × Education	Secondary/higher education (Yes vs. No) within Poor	0.97	0.68–1.40
Household wealth status × Education	Secondary/higher education (Yes vs. No) within Non-poor	1.12	0.82–1.54

AOR: Adjusted Odds Ratio; CI: Confidence Interval; Ref: Reference.

The outcome model was adjusted for age, sex, marital status, body mass index, ecological region, region of residence, and place of residence.

## Discussion

This study examined the association of household wealth and educational attainment with hypertension among adults in Lesotho using data from the 2023–2024 LDHS. Employing robust propensity score (PS)–based methods and accounting for the complex survey design, we found that adults from non-poor households had significantly higher odds of hypertension than their counterparts from poor households. The association was consistent across both PS matching and IPW approaches, and in sensitivity analyses using conventional multivariable logistic regression. In contrast, educational attainment was not independently associated with hypertension. Furthermore, the positive association between wealth and hypertension persisted across both education strata, suggesting that socioeconomic gradients in hypertension risk in Lesotho may be primarily driven by wealth-related lifestyle and contextual factors rather than education level alone.

The observed high prevalence of hypertension among underweight individuals in our study may reflect a combination of biological factors, including but not limited to chronic infectious or inflammatory conditions [29], early-life undernutrition [30], and prolonged exposure to psychosocial stressors and salt sensitivity [31]. These mechanisms may operate independently of BMI and therefore contribute to elevated blood pressure despite low body weight. This pattern may also reflect collider stratification bias [32,33], because stratifying on underweight status may lead to selection of individuals with underlying chronic illness associated with both weight loss and elevated blood pressure, thereby inflating the observed prevalence of hypertension in this subgroup. Our findings align with the literature from several sub-Saharan African settings where hypertension risk has been observed to increase with higher socioeconomic position. Studies from Ghana and South Africa reported that adults from wealthier households exhibited higher prevalence of hypertension, often attributed to urbanization, sedentary occupations, and increased consumption of calorie-dense foods [34,35]. Similar patterns have been reported in other LMICs experiencing epidemiological transition, where rising incomes and urban lifestyles contribute to the clustering of metabolic risk factors among higher socioeconomic groups [9,36].

On the other hand, in high-income countries, hypertension disproportionately affects individuals of lower socioeconomic status due to chronic stress, poor diet quality, and barriers to healthcare access [37,38]. These divergent patterns across contexts underscore the complex interplay between wealth, education, and cardiovascular risk as societies undergo demographic and nutritional transitions. In Lesotho, where rapid urbanization, dietary shifts, and obesity are increasingly prevalent, the direction of association observed in our analysis is consistent with the early phase of this transition [39–41].

The absence of a significant association between educational attainment and hypertension in our study is also noteworthy. Similar null associations were reported in Bangladesh [12], suggesting that education alone may not translate into healthier behaviors or better hypertension control in contexts where lifestyle risks are widespread across socioeconomic strata. In such contexts, cultural dietary practices, limited access to preventive health services, and restricted opportunities to apply health knowledge may attenuate the potential protective effect of education.

Several factors may explain the higher odds of hypertension among non-poor adults in Lesotho. First, wealthier individuals are more likely to reside in urban or peri-urban areas where exposure to processed foods, high sodium intake, and sedentary occupations are common [39–41]. Lesotho’s rapid economic and lifestyle changes, marked by increased availability of convenience foods, alcohol use, and reduced physical activity, mirror the broader regional trends in hypertension epidemiology [39–41]. Second, the association may reflect differential access to diagnosis rather than true differences in incidence: non-poor individuals may have better healthcare access and thus higher detection rates, potentially leading to an apparent socioeconomic gradient [42]. Third, psychosocial stress associated with economic competition, modernization, and urban crowding could also contribute to elevated blood pressure among the non-poor [43]. The persistence of this pattern across education strata suggests that material conditions, lifestyle factors, and contextual exposures tied to wealth may outweigh cognitive or informational advantages associated with formal education [44].

### Public health and policy implications

These findings suggest that hypertension prevention strategies in Lesotho should extend beyond traditionally targeted resource-constrained populations to include wealthier and urban groups that are increasingly exposed to lifestyle-related risk factors. Policy responses should address structural determinants of health, including urbanization, food environments, and access to healthy lifestyle options, alongside strengthening primary healthcare systems for equitable screening and treatment. Interventions targeting behavioral risk factors such as high salt intake, physical inactivity, and alcohol use are essential, particularly in rapidly urbanizing settings. In addition, strategies that consider psychosocial stress and the broader social context of economic transition may further enhance hypertension prevention efforts.

### Strengths and limitations

This study is among the first to apply propensity score-based causal methods to nationally representative data from Lesotho, enhancing covariate balance and reducing confounding in an observational setting. The use of complementary analytic approaches and the most recent LDHS data strengthens the robustness and relevance of the findings.

However, several limitations should be considered. First, the cross-sectional design precludes establishing temporality between exposures and hypertension, and therefore causal inferences should be interpreted with caution. In particular, the possibility of reverse causation cannot be ruled out, as underlying health conditions may influence both socioeconomic status and blood pressure. Second, blood pressure was measured at a single time point and may be subject to short-term variability or measurement error. Third, residual confounding due to unmeasured factors cannot be excluded. In particular, detailed behavioral variables such as dietary intake and physical activity were not available in the dataset. These factors are likely to lie on the causal pathway linking socioeconomic status to hypertension and, if included, could introduce overadjustment bias; however, their absence may also contribute to incomplete adjustment for relevant exposures. Despite these limitations, the consistency of findings across multiple analytic approaches supports the robustness of the observed associations.

## Conclusion

This nationally representative propensity score–based analysis demonstrates that household wealth is significantly associated with hypertension among adults in Lesotho. Non-poor individuals face a disproportionately higher burden of hypertension, independent of education and other sociodemographic factors. These findings underscore that as Lesotho undergoes socioeconomic and lifestyle transitions, hypertension may increasingly concentrate among the affluent, reflecting the early stages of the epidemiologic transition. Prevention and control strategies should therefore adopt a dual focus targeting both disadvantaged populations and wealthier urban groups through multisectoral policies that promote healthier environments, ensure equitable access to care, and strengthen population-wide blood pressure control.
