# Supplementary material for: Association of household wealth and education with hypertension among adults in Lesotho: Evidence from a propensity score–based study
Source: PLOS Glob Public Health. 2026 Jun 10;6(6):e0006599. doi: 10.1371/journal.pgph.0006599 (PMC13252731; doi:10.1371/journal.pgph.0006599)
Supplement: Attachment — Submitted filename: Response to reviewers.docx [file pgph.0006599.s002.docx]

**Submitted Title:** Association of household wealth and education with hypertension among adults in Lesotho: Evidence from a propensity score–based study

**Ref:** PGPH-D-26-00353

**RESPONSE TO EDITOR**

**JOURNAL REQUIREMENTS**

1. Please ensure that your Ethics Statement is available in its entirety at the beginning of your Methods section, under a subheading 'Ethics Statement'.

**Response:** Thank you for this important comment. We have revised the manuscript to include the full Ethics Statement at the beginning of the Methods section under the subheading “Ethics Statement,” in accordance with the journal’s guidelines.

2. If the reviewer comments include a recommendation to cite specific previously published works, please review and evaluate these publications to determine whether they are relevant and should be cited. There is no requirement to cite these works unless the editor has indicated otherwise.

**Response:** Thank you for this clarification..

3. Please review your reference list to ensure that it is complete and correct. If you have cited papers that have been retracted, please include the rationale for doing so in the manuscript text, or remove these references and replace them with relevant current references. Any changes to the reference list should be mentioned in the rebuttal letter that accompanies your revised manuscript. If you need to cite a retracted article, indicate the article’s retracted status in the References list and also include a citation and full reference for the retraction notice.

**Response:** Thank you for this important comment. We have carefully reviewed the reference list to ensure that all citations are accurate, complete, and up to date. No retracted articles were identified among the cited references, and all references have been retained or revised accordingly; any updates to the reference list have been incorporated in the revised manuscript.

**RESPONSE TO REVIEWERS**

**REVIEWER #1**

This study looked at the separate and combined relationships between adult hypertension in Lesotho and household wealth and educational attainment. My observations are given below:

(1) The study title is okay.

**Response to the reviewer:** Thank you for the comment!

(2) The abstract uses sound analytical techniques to address a significant public health issue. However, there is a lack of theoretical framework, the rationale behind the age restriction (18–49) is not entirely clear, and further discussion and integration of the implications for policy and psychological mechanisms underlying wealth–hypertension associations is necessary.

**Response to the reviewer:** Thank you for this important and insightful comment. We have revised the manuscript to strengthen the theoretical framework, clarify the age restriction, and enhance the discussion of policy implications and underlying mechanisms.

First, we have incorporated a conceptual framework in the Introduction to better contextualize the relationship between socioeconomic status and hypertension. Specifically, we now state: “Guided by established frameworks on social determinants of health, household wealth and educational attainment were conceptualized as distinct dimensions of socioeconomic status that influence hypertension through multiple pathways [18]. Household wealth reflects material resources and living conditions, shaping access to food environments, healthcare services, and patterns of urban residence, while educational attainment is associated with health literacy, behavioral capacity, and psychosocial resources such as perceived control and the ability to manage stress. These influences may operate through behavioral pathways (e.g., diet, physical activity, tobacco and alcohol use) and psychosocial processes, including chronic stress and social comparison, which can affect blood pressure through neuroendocrine mechanisms [18].

In the present study, this framework was operationalized using available LDHS covariates, including age, sex, marital status, body mass index (BMI), ecological zone, region of residence, and place of residence [12,19] (Fig 1). These variables were selected as potential confounders representing demographic, geographic, and anthropometric factors associated with both socioeconomic status and hypertension risk.

Although important behavioral determinants such as dietary intake and physical activity were not available in the dataset, these factors are likely to lie on the causal pathway linking socioeconomic status to hypertension and were therefore not explicitly modeled but are considered in the interpretation of the findings. Differences in access to healthcare and diagnosis may also contribute to observed disparities.

Within this framework, household wealth and educational attainment may operate both independently and jointly to influence hypertension risk. Their interaction may reflect how material resources and health-related knowledge combine within the sociocultural context of Lesotho to shape hypertension risk.”

Second, we have clarified the rationale for restricting the analysis to adults aged 18–49 years in the Study Population section. The manuscript now states: “Although individuals aged 15–17 years were eligible in the original DHS sample, we restricted the analysis to adults to ensure epidemiological and clinical comparability, as hypertension definitions, risk profiles, and physiological characteristics differ between adolescents and adults.”

Third, we have expanded the Discussion to better integrate policy implications and the mechanisms underlying the observed wealth–hypertension association. We have already discussed the higher prevalence of hypertension in Lesotho: “Several factors may explain the higher odds of hypertension among non-poor adults in Lesotho. First, wealthier individuals are more likely to reside in urban or peri-urban areas where exposure to processed foods, high sodium intake, and sedentary occupations are common [38–40]. Lesotho’s rapid economic and lifestyle changes, marked by increased availability of convenience foods, alcohol use, and reduced physical activity, mirror the broader regional trends in hypertension epidemiology [38–40]. Second, the association may reflect differential access to diagnosis rather than true differences in incidence: non-poor individuals may have better healthcare access and thus higher detection rates, potentially leading to an apparent socioeconomic gradient [41]. Third, psychosocial stress associated with economic competition, modernization, and urban crowding could also contribute to elevated blood pressure among the non-poor [42]. The persistence of this pattern across education strata suggests that material conditions, lifestyle factors, and contextual exposures tied to wealth may outweigh cognitive or informational advantages associated with formal education [43].”

In addition, we have strengthened the policy implications to emphasize structural interventions, including urban planning, food environments, equitable healthcare access, and context-specific strategies addressing behavioral risk factors and psychosocial stress: “These findings suggest that hypertension prevention strategies in Lesotho should extend beyond traditionally targeted resource-constrained populations to include wealthier and urban groups that are increasingly exposed to lifestyle-related risk factors. Policy responses should address structural determinants of health, including urbanization, food environments, and access to healthy lifestyle options, alongside strengthening primary healthcare systems for equitable screening and treatment. Interventions targeting behavioral risk factors such as high salt intake, physical inactivity, and alcohol use are essential, particularly in rapidly urbanizing settings. In addition, strategies that consider psychosocial stress and the broader social context of economic transition may further enhance hypertension prevention efforts.”

(3) The introduction is coherent and offers a socioeconomic and epidemiological justification. It does not, however, adequately incorporate psychological theory on SES-health pathways and reads primarily as a public health narrative (e.g. G. stress, behavior related to health, and perceived control). Instead of being critically synthesized, the literature review is descriptive. Propensity score methods could have a theoretical foundation for their justification. Additionally, describe how "interaction" is supposed to function in Lesotho's sociocultural context and make clear the conceptual model that guides hypotheses.

**Response to the reviewer:** Thanks! We have added the conceptual framework describing these: “Guided by established frameworks on social determinants of health, household wealth and educational attainment were conceptualized as distinct dimensions of socioeconomic status that influence hypertension through multiple pathways [18]. Household wealth reflects material resources and living conditions, shaping access to food environments, healthcare services, and patterns of urban residence, while educational attainment is associated with health literacy, behavioral capacity, and psychosocial resources such as perceived control and the ability to manage stress. These influences may operate through behavioral pathways (e.g., diet, physical activity, tobacco and alcohol use) and psychosocial processes, including chronic stress and social comparison, which can affect blood pressure through neuroendocrine mechanisms [18].

In the present study, this framework was operationalized using available LDHS covariates, including age, sex, marital status, body mass index (BMI), ecological zone, region of residence, and place of residence [12,19] (Fig 1). These variables were selected as potential confounders representing demographic, geographic, and anthropometric factors associated with both socioeconomic status and hypertension risk.

Although important behavioral determinants such as dietary intake and physical activity were not available in the dataset, these factors are likely to lie on the causal pathway linking socioeconomic status to hypertension and were therefore not explicitly modeled but are considered in the interpretation of the findings. Differences in access to healthcare and diagnosis may also contribute to observed disparities.

Within this framework, household wealth and educational attainment may operate both independently and jointly to influence hypertension risk. Their interaction may reflect how material resources and health-related knowledge combine within the sociocultural context of Lesotho to shape hypertension risk.”

(4) The techniques, which make use of complex survey adjustments and nationally representative Demographic and Health Survey data, are generally rigorous and well-described. Procedures for measuring blood pressure and defining hypertension are standard. However, considering the possibility of information loss, the argument for drawing a distinction between wealth and education needs to be reinforced. Describe the integration of survey design features into the estimation and matching of propensity scores. When wealth and education are treated as exposures alternately, including them in PS covariates runs the risk of causing overadjustment. Cross-sectional limitations and residual confounding need to be fully acknowledged.

**Response to the reviewer:**

Thank you for this thoughtful and comprehensive comment. We have revised the manuscript to address each of the concerns raised.

First, we have strengthened the conceptual justification for distinguishing between household wealth and educational attainment. While both are indicators of socioeconomic status, they capture distinct dimensions. Household wealth reflects material resources and living conditions, which influence environmental exposures and access to healthcare, whereas educational attainment represents cognitive and psychosocial resources, including health literacy, perceived control, and the ability to adopt health-promoting behaviors. Treating these variables separately allows for a more nuanced assessment of their independent and joint associations with hypertension and reduces the risk of masking heterogeneous pathways.

We added this in the conceptual framework as following: “Guided by established frameworks on social determinants of health, household wealth and educational attainment were conceptualized as distinct dimensions of socioeconomic status that influence hypertension through multiple pathways [18]. Household wealth reflects material resources and living conditions, shaping access to food environments, healthcare services, and patterns of urban residence, while educational attainment is associated with health literacy, behavioral capacity, and psychosocial resources such as perceived control and the ability to manage stress. These influences may operate through behavioral pathways (e.g., diet, physical activity, tobacco and alcohol use) and psychosocial processes, including chronic stress and social comparison, which can affect blood pressure through neuroendocrine mechanisms [18].”

Second, we have clarified the integration of survey design features into the propensity score analyses. Specifically, propensity scores were estimated while accounting for survey design variables (strata and cluster), and stabilized inverse probability weights were combined with sampling weights to generate population-representative estimates. In addition, all outcome models were estimated using survey-adjusted (“svy”) procedures to appropriately account for the complex sampling design. We have added this in the statistical analysis section: “All statistical analyses were conducted by Stata 19.0 (StataCorp LLC, College Station, TX). All analyses accounted for the complex survey design, including sampling weights, clusters, and strata, using the “svy” command of Stata. The sample characteristics were first described using weighted percentages and assessed bivariate associations between covariates and hypertension with Rao-Scott Chi-square tests adjusted for design effects [24].

For the main analysis, we applied propensity score (PS) methods to estimate the association of household wealth and education with hypertension [25]. Separate PS models were constructed for each exposure. When household wealth status was treated as the exposure, educational attainment was included as a covariate in the PS model. Conversely, when educational attainment was treated as the exposure, household wealth status was included as a covariate. In both cases, PS were estimated using a logistic model incorporating age, sex, marital status, body mass index, ecological zone, region of residence, and place of residence, along with survey design variables (strata and cluster), to control for confounding.”

Third, regarding the potential for overadjustment, we clarified that separate propensity score models were constructed for each exposure. When household wealth status was treated as the exposure, educational attainment was included as a covariate, and vice versa. This approach was adopted because wealth and education represent distinct socioeconomic dimensions rather than mediators of one another. Therefore, their inclusion in alternate models is intended to control for confounding rather than induce overadjustment. We included in this in the statistical analysis section: “For the main analysis, we applied propensity score (PS) methods to estimate the association of household wealth and education with hypertension [25]. Separate PS models were constructed for each exposure. When household wealth status was treated as the exposure, educational attainment was included as a covariate in the PS model. Conversely, when educational attainment was treated as the exposure, household wealth status was included as a covariate. In both cases, PS were estimated using a logistic model incorporating age, sex, marital status, body mass index, ecological zone, region of residence, and place of residence, along with survey design variables (strata and cluster), to control for confounding.”

Finally, we have strengthened the limitations section to more fully acknowledge the cross-sectional nature of the data and the potential for residual confounding. Specifically, we note the inability to establish temporality, the possibility of reverse causation, and the absence of certain behavioral variables (e.g., diet and physical activity), which may contribute to unmeasured confounding. These limitations have now been explicitly discussed in the revised manuscript. We have revised the limitation section as following: “However, several limitations should be considered. First, the cross-sectional design precludes establishing temporality between exposures and hypertension, and therefore causal inferences should be interpreted with caution. In particular, the possibility of reverse causation cannot be ruled out, as underlying health conditions may influence both socioeconomic status and blood pressure. Second, blood pressure was measured at a single time point and may be subject to short-term variability or measurement error. Third, residual confounding due to unmeasured factors cannot be excluded. In particular, detailed behavioral variables such as dietary intake and physical activity were not available in the dataset. These factors are likely to lie on the causal pathway linking socioeconomic status to hypertension and, if included, could introduce overadjustment bias; however, their absence may also contribute to incomplete adjustment for relevant exposures. Despite these limitations, the consistency of findings across multiple analytic approaches supports the robustness of the observed associations.”

(5) The findings, especially the application of propensity score matching and weighting to address confounding, are generally well-presented and analytically rigorous. The remarkably high prevalence of hypertension among underweight people (48.6%) is surprising, though, and calls for more research or sensitivity testing. A contextual discussion of Lesotho's urbanization and lifestyle factors would be beneficial for the interpretation of wealth gradients. Furthermore, in descriptive comparisons, providing effect sizes in addition to p-values may enhance the substantive interpretation beyond statistical significance.

**Response to the reviewer:** Thank you for this thoughtful and constructive comment. We have addressed each of the points raised.

First, we carefully re-examined the prevalence of hypertension among underweight individuals (48.6%) and confirm that this estimate is correct. We agree that this finding is unexpected and warrants cautious interpretation.

To address this, we have expanded the Discussion to provide potential explanations. First, this finding may reflect reverse causation, where individuals with underlying chronic illness or frailty experience weight loss while also having elevated blood pressure. Second, the estimate may be influenced by small subgroup size and instability, which can lead to exaggerated prevalence estimates. Third, measurement variability or residual confounding may also contribute to this observation.

We have revised the Discussion to explicitly acknowledge these possibilities and to emphasize that this finding should be interpreted with caution and warrants further investigation in longitudinal studies.

We have added this in the discussion section as following “The observed high prevalence of hypertension among underweight individuals in our study may reflect a combination of biological factors, including but not limited to chronic infectious or inflammatory conditions [29], early-life undernutrition [30], and prolonged exposure to psychosocial stressors and salt sensitivity [31]. These mechanisms may operate independently of BMI and therefore contribute to elevated blood pressure despite low body weight. This pattern may also reflect collider stratification bias [32,33], because stratifying on underweight status may lead to selection of individuals with underlying chronic illness associated with both weight loss and elevated blood pressure, thereby inflating the observed prevalence of hypertension in this subgroup.”

Second, we already discussed Lesotho's urbanization and lifestyle factors in the discussion section: “Several factors may explain the higher odds of hypertension among non-poor adults in Lesotho. First, wealthier individuals are more likely to reside in urban or peri-urban areas where exposure to processed foods, high sodium intake, and sedentary occupations are common [38–40]. Lesotho’s rapid economic and lifestyle changes, marked by increased availability of convenience foods, alcohol use, and reduced physical activity, mirror the broader regional trends in hypertension epidemiology [38–40]. Second, the association may reflect differential access to diagnosis rather than true differences in incidence: non-poor individuals may have better healthcare access and thus higher detection rates, potentially leading to an apparent socioeconomic gradient [41]. Third, psychosocial stress associated with economic competition, modernization, and urban crowding could also contribute to elevated blood pressure among the non-poor [42]. The persistence of this pattern across education strata suggests that material conditions, lifestyle factors, and contextual exposures tied to wealth may outweigh cognitive or informational advantages associated with formal education [43].”

Third, we have already discussed effect sizes in addition to p-values in the descriptive comparisons.

(6) The discussion is generally well-reasoned and contextualized within the literature of LMICs and sub-Saharan Africa. Nevertheless, causal language (e.g. A. Given the cross-sectional design and potential residual confounding, "driven by" should be further moderated. In particular, the measurement of hypertension in LDHS warrants further elaboration of the differential diagnosis versus detection explanation. Consideration of alternative mechanisms and null education findings more critically (e.g., health literacy level) would improve understanding. Lastly, policy implications could strengthen the distinction between structural interventions and short-term clinical strategies.

**Response to the reviewer:** Thank you for this thoughtful comment. We have revised the Discussion to moderate causal language, expand interpretation of the findings, and strengthen the policy implications.

First, we have softened causal phrasing throughout the Discussion. Terms such as “driven by” have been replaced with more appropriate language for cross-sectional analyses (e.g., “associated with,” “may reflect,” or “may be explained by”) to avoid implying causality.

Second, we have clarified that although the LDHS hypertension definition includes self-reported diagnosis and medication use, it also relies on standardized blood pressure measurements. Therefore, differential diagnosis alone is unlikely to fully explain the observed wealth gradient. We now discuss detection as one possible contributing factor, while emphasizing that measured blood pressure differences and wealth-related lifestyle or contextual exposures may also underlie the association. We added the following statement in the discussion: “Second, the association may partly reflect differential access to healthcare and diagnosis, as individuals from non-poor households may have greater access to screening and treatment services. However, hypertension in the LDHS was also defined using standardized blood pressure measurements, in addition to self-reported diagnosis and medication use. Therefore, the observed wealth gradient is unlikely to be explained solely by differential detection and may also reflect differences in lifestyle, urban residence, adiposity, and other contextual exposures [39].”

(7) The references are ok. Please correct it by following the journal’s guidelines. The tables and figures should be prepared following standard guidelines.

**Response to the reviewer:** Thank you for this comment. We have revised the references, tables, and figures to ensure full compliance with the journal’s formatting and presentation guidelines, improving consistency and clarity throughout the manuscript.

**REVIEWER #2**

1. (Abstract): Please specify that the 21.2% hypertension prevalence reported in the abstract is weighted, to ensure consistency with the Methods.

**Response to the reviewer:** Thank you for this comment. We have revised the abstract to clarify that the reported hypertension prevalence (21.2%) represents weighted estimates, ensuring consistency with the Methods section.

The revised sentence now reads: “Overall, 21.2% (weighted) of participants were hypertensive.”

2. (Methods): Please explain why participants aged 15–17 years were excluded from the analysis, despite being eligible in the original DHS sample.

**Response to the reviewer:** Thank you for this important comment. We restricted the analysis to the adult population (18–49 years) to ensure epidemiological and clinical comparability. Although individuals aged 15–17 years were included in the original DHS sample, they were excluded from this analysis because hypertension definitions, risk profiles, and physiological characteristics differ between adolescents and adults. Including adolescents could introduce heterogeneity and potential misclassification, thereby affecting the validity and interpretability of the findings. Therefore, focusing on adults allowed for a more consistent and meaningful assessment of the association between socioeconomic factors and hypertension.

We added the following section in the manuscript: “Although individuals aged 15–17 years were eligible in the original DHS sample, we restricted the analysis to adults to ensure epidemiological and clinical comparability, as hypertension definitions, risk profiles, and physiological characteristics differ between adolescents and adults.”

3. (Methods): There is an inconsistency in covariate reporting: “working status” appears in the adjustment notes for Tables 2–4, but is not described in the Methods. Please clarify whether it was included in the models

**Response to the reviewer:** Thank you for highlighting this inconsistency. This was a typographical error, and we apologize for the oversight. “Working status” was not included as a covariate in any of the models. We have revised the adjustment notes in Tables 2–4 to ensure consistency with the Methods section.

4. (Methods): There is an inconsistency in the description of the propensity score models. Household wealth status and educational attainment are described as exposures, but later also appear among the PS covariates. Please clarify the PS specification for each exposure separately.

**Response to the reviewer:** Thank you for this important observation. We have clarified the specification of the propensity score (PS) models in the Methods section. Separate PS models were constructed for each exposure. When household wealth status was treated as the exposure, educational attainment was included as a covariate in the PS model. Conversely, when educational attainment was treated as the exposure, household wealth status was included as a covariate. In both cases, the PS models additionally included age, sex, marital status, body mass index, ecological zone, region of residence, place of residence, and survey design variables (strata and cluster). This approach was used to control for confounding, as wealth and education represent distinct dimensions of socioeconomic status rather than mediators of one another. The Methods section has been revised to clearly reflect this specification.

We added the following section in the methods section in the revised manuscript: “Separate PS models were constructed for each exposure. When household wealth status was treated as the exposure, educational attainment was included as a covariate in the PS model. Conversely, when educational attainment was treated as the exposure, household wealth status was included as a covariate.”

5. (Results): The reported hypertension prevalence in the underweight group (48.6%) appears unusually high and warrants verification, as well as further explanation in the Discussion

**Response to the reviewer:** Thank you for this important observation. We have carefully rechecked the data and confirm that the reported hypertension prevalence among underweight individuals (48.6%) is correct. We agree that this estimate appears unusually high and warrants cautious interpretation.

To address this, we have expanded the Discussion to provide potential explanations. First, this finding may reflect reverse causation, where individuals with underlying chronic illness or frailty experience weight loss while also having elevated blood pressure. Second, the estimate may be influenced by small subgroup size and instability, which can lead to exaggerated prevalence estimates. Third, measurement variability or residual confounding may also contribute to this observation.

We have revised the Discussion to explicitly acknowledge these possibilities and to emphasize that this finding should be interpreted with caution and warrants further investigation in longitudinal studies.

We have added this in the discussion section as following “The observed high prevalence of hypertension among underweight individuals in our study may reflect a combination of biological factors, including but not limited to chronic infectious or inflammatory conditions [29], early-life undernutrition [30], and prolonged exposure to psychosocial stressors and salt sensitivity [31]. These mechanisms may operate independently of BMI and therefore contribute to elevated blood pressure despite low body weight. This pattern may also reflect collider stratification bias [32,33], because stratifying on underweight status may lead to selection of individuals with underlying chronic illness associated with both weight loss and elevated blood pressure, thereby inflating the observed prevalence of hypertension in this subgroup.”

6. (Table 1): The presentation of Table 1 is unclear because unweighted frequencies and weighted percentages are shown together under “N (%)”. Please relabel the columns or separate unweighted counts from weighted estimates.

**Response to the reviewer:** Thank you for this important comment. We agree that presenting unweighted frequencies and weighted percentages within a single column may cause confusion. We have revised Table 1 to clearly distinguish between unweighted counts and weighted percentages by separating them into different columns. Specifically, we now present unweighted frequencies (N) and weighted percentages (%) in separate columns, and we have updated the table title and footnote accordingly to improve clarity and consistency.

7. I suggest that the authors consider citing the article (<https://doi.org/10.52225/narra.v5i1.1759>), as it appears relevant to the discussion of hypertension determinants and may help situate the observed associations with household wealth and education in a broader epidemiological context

**Response to the reviewer:** Thank you for this helpful suggestion. We carefully reviewed the recommended article. While it provides valuable insights into the molecular and biological mechanisms underlying hypertension, particularly involving microRNAs and the renin-angiotensin system, its focus is not directly aligned with the primary objective of our study, which examines socioeconomic determinants of hypertension.

Given this difference in scope, we did not include this reference in the revised manuscript. However, we appreciate the suggestion and acknowledge the importance of biological pathways in the broader context of hypertension research.
