# Supplementary material for: Association of household wealth and education with hypertension among adults in Lesotho: Evidence from a propensity score–based study
Source: PLOS Glob Public Health. 2026 Jun 10;6(6):e0006599. doi: 10.1371/journal.pgph.0006599 (PMC13252731; doi:10.1371/journal.pgph.0006599)
Supplement: Peer review history

# Peer review: Decision Letter 0

**DOI:** 10.1371/journal.pgph.0006599.r001

**Authors:** Muhammad Iqhrammullah

22 Apr 2026

PGPH-D-26-00353

Association of household wealth and education with hypertension among adults in Lesotho: Evidence from a propensity score–based study

PLOS Global Public Health

Dear Dr. Gupta,

Thank you for submitting your manuscript to PLOS Global Public Health. After careful consideration, we feel that it has merit but does not fully meet PLOS Global Public Health’s publication criteria as it currently stands. Therefore, we invite you to submit a revised version of the manuscript that addresses the points raised during the review process.

Please submit your revised manuscript by May 22 2026 11:59PM. If you will need more time than this to complete your revisions, please reply to this message or contact the journal office at globalpubhealth@plos.org. When you're ready to submit your revision, log on to https://www.editorialmanager.com/pgph/ and select the 'Submissions Needing Revision' folder to locate your manuscript file.

Please include the following items when submitting your revised manuscript:

- A letter that responds to each point raised by the editor and reviewer(s). You should upload this letter as a separate file labeled 'Response to Reviewers'.
- A marked-up copy of your manuscript that highlights changes made to the original version. You should upload this as a separate file labeled 'Revised Manuscript with Track Changes'.
- An unmarked version of your revised paper without tracked changes. You should upload this as a separate file labeled 'Manuscript'.

Guidelines for resubmitting your figure files are available below the reviewer comments at the end of this letter.

As the corresponding author, your ORCID iD is verified in the submission system and will appear in the published article. PLOS supports the use of ORCID, and we encourage all coauthors to register for an ORCID iD and use it as well. Please encourage your coauthors to verify their ORCID iD within the submission system before final acceptance, as unverified ORCID iDs will not appear in the published article. *Only* the individual author can complete the verification step; PLOS staff *cannot* verify ORCID iDs on behalf of authors.

We look forward to receiving your revised manuscript.

Kind regards,

Muhammad Iqhrammullah, Ph.D

Academic Editor

PLOS Global Public Health

**Journal Requirements:**

1. Please ensure that your Ethics Statement is available in its entirety at the beginning of your Methods section, under a subheading 'Ethics Statement'.

If the reviewer comments include a recommendation to cite specific previously published works, please review and evaluate these publications to determine whether they are relevant and should be cited. There is no requirement to cite these works unless the editor has indicated otherwise.

Please review your reference list to ensure that it is complete and correct. If you have cited papers that have been retracted, please include the rationale for doing so in the manuscript text, or remove these references and replace them with relevant current references. Any changes to the reference list should be mentioned in the rebuttal letter that accompanies your revised manuscript. If you need to cite a retracted article, indicate the article’s retracted status in the References list and also include a citation and full reference for the retraction notice.

**Additional Editor Comments (if provided):**

[Note: HTML markup is below. Please do not edit.]

Reviewers' comments:

Reviewer's Responses to Questions

**Comments to the Author**

1. Does this manuscript meet PLOS Global Public Health’s publication criteria? Is the manuscript technically sound, and do the data support the conclusions? The manuscript must describe methodologically and ethically rigorous research with conclusions that are appropriately drawn based on the data presented.

Reviewer #1: Yes

Reviewer #2: Partly

**********

2. Has the statistical analysis been performed appropriately and rigorously?

Reviewer #1: Yes

Reviewer #2: No

**********

3. Have the authors made all data underlying the findings in their manuscript fully available (please refer to the Data Availability Statement at the start of the manuscript PDF file)?

The PLOS Data policy requires authors to make all data underlying the findings described in their manuscript fully available without restriction, with rare exception. The data should be provided as part of the manuscript or its supporting information, or deposited to a public repository. For example, in addition to summary statistics, the data points behind means, medians and variance measures should be available. If there are restrictions on publicly sharing data—e.g. participant privacy or use of data from a third party—those must be specified.

Reviewer #1: Yes

Reviewer #2: Yes

**********

4. Is the manuscript presented in an intelligible fashion and written in standard English?

PLOS Global Public Health does not copyedit accepted manuscripts, so the language in submitted articles must be clear, correct, and unambiguous. Any typographical or grammatical errors should be corrected at revision, so please note any specific errors here.

Reviewer #1: Yes

Reviewer #2: Yes

**********

5. Review Comments to the Author

Please use the space provided to explain your answers to the questions above. You may also include additional comments for the author, including concerns about dual publication, research ethics, or publication ethics. (Please upload your review as an attachment if it exceeds 20,000 characters)

**Reviewer #1:** This study looked at the separate and combined relationships between adult hypertension in Lesotho and household wealth and educational attainment. My observations are given below:

(1) The study title is okay.

(2) The abstract uses sound analytical techniques to address a significant public health issue. However, there is a lack of theoretical framework, the rationale behind the age restriction (18–49) is not entirely clear, and further discussion and integration of the implications for policy and psychological mechanisms underlying wealth–hypertension associations is necessary.

(3) The introduction is coherent and offers a socioeconomic and epidemiological justification. It does not, however, adequately incorporate psychological theory on SES-health pathways and reads primarily as a public health narrative (e.g. G. stress, behavior related to health, and perceived control). Instead of being critically synthesized, the literature review is descriptive. Propensity score methods could have a theoretical foundation for their justification. Additionally, describe how "interaction" is supposed to function in Lesotho's sociocultural context and make clear the conceptual model that guides hypotheses.

(4) The techniques, which make use of complex survey adjustments and nationally representative Demographic and Health Survey data, are generally rigorous and well-described. Procedures for measuring blood pressure and defining hypertension are standard. However, considering the possibility of information loss, the argument for drawing a distinction between wealth and education needs to be reinforced. Describe the integration of survey design features into the estimation and matching of propensity scores. When wealth and education are treated as exposures alternately, including them in PS covariates runs the risk of causing overadjustment. Cross-sectional limitations and residual confounding need to be fully acknowledged.

(5) The findings, especially the application of propensity score matching and weighting to address confounding, are generally well-presented and analytically rigorous. The remarkably high prevalence of hypertension among underweight people (48.6%) is surprising, though, and calls for more research or sensitivity testing. A contextual discussion of Lesotho's urbanization and lifestyle factors would be beneficial for the interpretation of wealth gradients. Furthermore, in descriptive comparisons, providing effect sizes in addition to p-values may enhance the substantive interpretation beyond statistical significance.

(6) The discussion is generally well-reasoned and contextualized within the literature of LMICs and sub-Saharan Africa. Nevertheless, causal language (e.g. A. Given the cross-sectional design and potential residual confounding, "driven by" should be further moderated. In particular, the measurement of hypertension in LDHS warrants further elaboration of the differential diagnosis versus detection explanation. Consideration of alternative mechanisms and null education findings more critically (e.g., health literacy level) would improve understanding. Lastly, policy implications could strengthen the distinction between structural interventions and short-term clinical strategies.

(7) The references are ok. Please correct it by following the journal’s guidelines. The tables and figures should be prepared following standard guidelines.

**Reviewer #2:** 1. (Abstract): Please specify that the 21.2% hypertension prevalence reported in the abstract is weighted, to ensure consistency with the Methods.

2. (Methods): Please explain why participants aged 15–17 years were excluded from the analysis, despite being eligible in the original DHS sample.

3. (Methods): There is an inconsistency in covariate reporting: “working status” appears in the adjustment notes for Tables 2–4, but is not described in the Methods. Please clarify whether it was included in the models

4. (Methods): There is an inconsistency in the description of the propensity score models. Household wealth status and educational attainment are described as exposures, but later also appear among the PS covariates. Please clarify the PS specification for each exposure separately.

5. (Results): The reported hypertension prevalence in the underweight group (48.6%) appears unusually high and warrants verification, as well as further explanation in the Discussion

6. (Table 1): The presentation of Table 1 is unclear because unweighted frequencies and weighted percentages are shown together under “N (%)”. Please relabel the columns or separate unweighted counts from weighted estimates.

7. I suggest that the authors consider citing the article (https://doi.org/10.52225/narra.v5i1.1759), as it appears relevant to the discussion of hypertension determinants and may help situate the observed associations with household wealth and education in a broader epidemiological context

**********

6. PLOS authors have the option to publish the peer review history of their article (what does this mean?). If published, this will include your full peer review and any attached files.

**Do you want your identity to be public for this peer review?** If you choose “no”, your identity will remain anonymous but your review may still be made public.

For information about this choice, including consent withdrawal, please see our Privacy Policy.

Reviewer #1: **Yes:** Gyanesh Kumar Tiwari

Reviewer #2: No

**********

[NOTE: If reviewer comments were submitted as an attachment file, they will be attached to this email and accessible via the submission site. Please log into your account, locate the manuscript record, and check for the action link "View Attachments". If this link does not appear, there are no attachment files.]

**Figure Resubmissions:**

While revising your submission, we strongly recommend that you use PLOS’s NAAS tool (https://ngplosjournals.pagemajik.ai/artanalysis) to test your figure files. NAAS can convert your figure files to the TIFF file type and meet basic requirements (such as print size, resolution), or provide you with a report on issues that do not meet our requirements and that NAAS cannot fix.

After uploading your figures to PLOS’s NAAS tool - https://ngplosjournals.pagemajik.ai/artanalysis, NAAS will process the files provided and display the results in the "Uploaded Files" section of the page as the processing is complete. If the uploaded figures meet our requirements (or NAAS is able to fix the files to meet our requirements), the figure will be marked as "fixed" above. If NAAS is unable to fix the files, a red "failed" label will appear above. When NAAS has confirmed that the figure files meet our requirements, please download the file via the download option, and include these NAAS processed figure files when submitting your revised manuscript.

# Author comment: Author response to Decision Letter 1

**DOI:** 10.1371/journal.pgph.0006599.r002

5 May 2026

Attachment Submitted filename: Response to reviewers.docx

# Peer review: Decision Letter 1

**DOI:** 10.1371/journal.pgph.0006599.r003

**Authors:** Muhammad Iqhrammullah

18 May 2026

Association of household wealth and education with hypertension among adults in Lesotho: Evidence from a propensity score–based study

PGPH-D-26-00353R1

Dear Dr. Gupta,

We are pleased to inform you that your manuscript 'Association of household wealth and education with hypertension among adults in Lesotho: Evidence from a propensity score–based study' has been provisionally accepted for publication in PLOS Global Public Health.

Before your manuscript can be formally accepted you will need to complete some formatting changes, which you will receive in a follow up email. A member of our team will be in touch with a set of requests.

Please note that your manuscript will not be scheduled for publication until you have made the required changes, so a swift response is appreciated.

IMPORTANT: The editorial review process is now complete. PLOS will only permit corrections to spelling, formatting or significant scientific errors from this point onwards. Requests for major changes, or any which affect the scientific understanding of your work, will cause delays to the publication date of your manuscript.

If your institution or institutions have a press office, please notify them about your upcoming paper to help maximize its impact. If they'll be preparing press materials, please inform our press team as soon as possible -- no later than 48 hours after receiving the formal acceptance. Your manuscript will remain under strict press embargo until 2 pm Eastern Time on the date of publication. For more information, please contact globalpubhealth@plos.org.

Thank you again for supporting Open Access publishing; we are looking forward to publishing your work in PLOS Global Public Health.

Best regards,

Muhammad Iqhrammullah, Ph.D

Academic Editor

PLOS Global Public Health

***********************************************************

Reviewer Comments (if any, and for reference):

Reviewer's Responses to Questions

**Comments to the Author**

1. If the authors have adequately addressed your comments raised in a previous round of review and you feel that this manuscript is now acceptable for publication, you may indicate that here to bypass the “Comments to the Author” section, enter your conflict of interest statement in the “Confidential to Editor” section, and submit your "Accept" recommendation.

Reviewer #1: All comments have been addressed

Reviewer #2: All comments have been addressed

**********

2. Does this manuscript meet PLOS Global Public Health’s publication criteria? Is the manuscript technically sound, and do the data support the conclusions? The manuscript must describe methodologically and ethically rigorous research with conclusions that are appropriately drawn based on the data presented.

Reviewer #1: Yes

Reviewer #2: Yes

**********

3. Has the statistical analysis been performed appropriately and rigorously?

Reviewer #1: Yes

Reviewer #2: Yes

**********

4. Have the authors made all data underlying the findings in their manuscript fully available (please refer to the Data Availability Statement at the start of the manuscript PDF file)?

The PLOS Data policy requires authors to make all data underlying the findings described in their manuscript fully available without restriction, with rare exception. The data should be provided as part of the manuscript or its supporting information, or deposited to a public repository. For example, in addition to summary statistics, the data points behind means, medians and variance measures should be available. If there are restrictions on publicly sharing data—e.g. participant privacy or use of data from a third party—those must be specified.

Reviewer #1: Yes

Reviewer #2: Yes

**********

5. Is the manuscript presented in an intelligible fashion and written in standard English?

PLOS Global Public Health does not copyedit accepted manuscripts, so the language in submitted articles must be clear, correct, and unambiguous. Any typographical or grammatical errors should be corrected at revision, so please note any specific errors here.

Reviewer #1: Yes

Reviewer #2: Yes

**********

6. Review Comments to the Author

Please use the space provided to explain your answers to the questions above. You may also include additional comments for the author, including concerns about dual publication, research ethics, or publication ethics. (Please upload your review as an attachment if it exceeds 20,000 characters)

Reviewer #1: I read the manuscript again. I believe all the queries raised during the initial review have been satisfactorily addressed by the authors. The manuscript is now more readable and comprehensive. It fits more precisely now. I think the manuscript as it now has the potential to advance knowledge.

Reviewer #2: I have no further comments. The manuscript has been strengthened through the review process, and all previous comments have been well addressed. I recommend acceptance

**********

7. PLOS authors have the option to publish the peer review history of their article (what does this mean?). If published, this will include your full peer review and any attached files.

**Do you want your identity to be public for this peer review?** If you choose “no”, your identity will remain anonymous but your review may still be made public.

For information about this choice, including consent withdrawal, please see our Privacy Policy.

Reviewer #1: **Yes:** Gyanesh Kumar Tiwari

Reviewer #2: No

**********
